# Exploring the ethical, legal, and social implications of cybernetic avatars

**DOI:** 10.3389/frobt.2025.1724149

**Published:** 2026-01-05

**Authors:** Ryuma Shineha

**Affiliations:** Graduate School of Media Design, Keio University, Yokohama, Japan

**Keywords:** cybernetic avatar, ethical, legal, and social implications (ELSI), information and communication technology, responsible research and innovation (RRI), robotics

## Abstract

A cybernetic avatar (CA) is a concept that encompasses not only avatars representing virtual bodies in cyberspace but also information and communication technology (ICT) and robotic technologies that enhance the physical, cognitive, and perceptual capabilities of humans. CAs can enable multiple people to remotely operate numerous avatars and robots together to perform complex tasks on a large scale and create the necessary infrastructure for their operation and other related activities. However, due to the novelty of this concept, the ethical, legal, and social implications (ELSI) of CAs have not been discussed sufficiently. Therefore, the objective of this paper is to provide an overview of ELSI in the context of a CA, taking into account the implications from fields similar to that of CAs, such as robotic avatars, virtual avatars, metaverses, virtual reality, extended reality, social robots, human–robot interaction, telepresence, telexistence, embodied technology, and exoskeletons. In our review of ELSI in related fields, we found common themes: safety and security, data privacy, identity theft and identity loss, manipulation, intellectual property management, user addiction and overdependence, cyber abuse, risk management in a specific domain (e.g., medical applications), regulatory gaps, balance between free expression and harmful content, accountability, transparency, distributive justice, prevention of inequalities, dual use, and conceptual changes of familiarity. These issues should not be ignored when considering the social implementation of CAs.

## 1 Introduction

A cybernetic avatar (CA) is a concept that encompasses not only avatars that represent virtual bodies in cyberspace but also information and communication technology (ICT) and robotics technologies that enhance the physical, cognitive, and perceptual capabilities of humans. CAs can enable multiple people to operate numerous avatars and robots together remotely to perform complex tasks on a large scale. In Japan, CA research and development has been actively pursued as part of the Japanese government’s major funding program, “Moonshot Program Goal 1.”

Ishiguro describes a CA as a new communication medium that frees people from physical limitations and enables them to work freely. The purpose of the CA is also discussed as follows:

Through CAs, people with disabilities and the elderly will gain new opportunities for social engagement. Moreover, we envision a more inclusive society where humans coexist with robots and AI, fostering diversity and collaboration (Ishiguro, 2025).

Contextualizing emerging science and technologies in society has become an important issue in today’s knowledge-based society. A central concern is how to maximize the benefits and minimize the potential risks of emerging sciences and technologies. This issue is primarily about understanding and addressing the ethical, legal, and social implications (ELSI) as early as possible. However, the ELSI of CA has not yet been sufficiently discussed due to the novelty of this concept. Although several recent studies have focused on CAs, more details about possible ELSI still need to be investigated. The ELSI of a CA has been influenced not only by AI but also by related fields such as robotic avatars, virtual avatars, metaverses, virtual reality/extended reality (VR/XR), social robots, HRI, telepresence, telexistence, embodied technology, and exoskeletons. Therefore, the objective of this study is to review the ELSI in related fields and anticipate the ELSI of CA.

**FIGURE 1:**
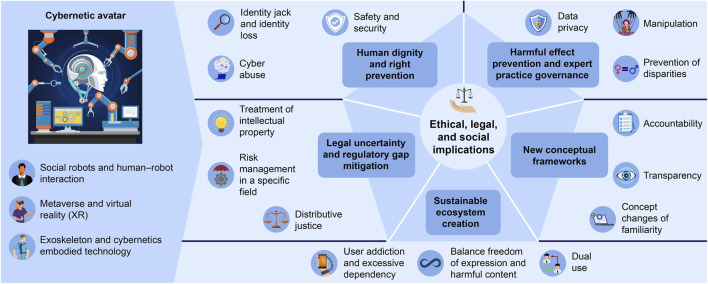
Overview of the ELSI concerning Cybernetic Avatar.

This analysis is intended to provide a minimal overview of ELSI in relation to a CA (Figure 1). Thus, it does not necessarily guarantee complete coverage and is only an interim report. However, it is unlikely that the ELSI of CAs can be discussed without considering the perspectives and issues raised in this study. Furthermore, given the fact that similar issues continue to emerge, it is difficult to discuss the ELSI of CAs without considering the ELSI highlighted in this study. It is hoped that this paper will serve as a useful reference for future discussions on ELSI and governance in the context of CAs.

## 2 ELSI concerning social robot and human robot interaction

In this section, implications from the discussions on social robots and HRI have been drawn out. With the rapid development of robots and AI, expectations from social robotics and HRI have increased. Many devices and robots have already been implemented in healthcare, education, sales, entertainment, and so on. They have become everyday agents in our lives. Although the public perception of social assistive robots (SARs) is generally positive, a previous study has shown that public attitudes are influenced by a sense of anxiety, acceptance, and trust. The acceptance of SAR has increased in medical and care applications (Naneva et al., 2020). As Torras discussed “as usual, the technology is developing faster than its agreed-upon regulation, and several organizations and associations have felt the urgent need to develop specific standards and guidelines for their particular domains of activity” (Torras, 2024). It is therefore important to consider ELSI and its guidelines (Fosch-Villaronga et al., 2020).

Torras analyzed ELSI’s concerns about social robots in two categories: individual and social (Torras, 2024). At the individual level, human dignity, autonomy, robot transparency, emotional attachment, privacy, and safety are important concerns. An important aspect of human dignity is that robots must not engage in behaviors that disrespect the user or interfere with autonomy, especially in situations such as elderly care. Human autonomy, which focuses on design, must avoid manipulation or overdependence, particularly through persuasive “nudges.” This point can be connected to the concerns about deception by robots (Wullenkord and Eyssel, 2020). In particular, we can focused on the empathy effect that convenient deception by robots has on users, from the perspective of vulnerability mirroring Robot transparency is the view that robots must not deceptively mimic emotions or intelligence and that users should understand their mechanical nature. Furthermore, there are concerns that emotional attachments to robots may lead to social isolation or psychological harm. It was pointed out that privacy and safety are interpreted as risks, such as data misuse, surveillance, and hacking, which need to be addressed appropriately, especially in domestic environments. Torras also refers to social-level concerns. In terms of justice, the use of robotic services should be fair. It is necessary to avoid the digital divide and algorithmic bias (Torras, 2024). Concerns for freedom require that we consider surveillance, loss of negative freedom (e.g., coercion), and “libertarian paternalism.” In addition, concerns about responsibility were highlighted as designers and policymakers share responsibility for ensuring that robotic systems are sustainable, inclusive, and transparent.

It is easy to understand that these concerns will be more serious in sensitive fields such as medicine, rehabilitation, and care (Boada et al., 2021; Niemelä et al., 2021). They summarized six themes were identified in relation to care: loss of human contact, legitimacy of introducing SARs, quality of practice, human moral practice, trust, impact on the concept of care, and role disruption. These issues pertain to care practices. What constitutes appropriate care for people? How can SARs provide appropriate care? Is there a risk that trust—the most important factor in care—will break down between patients and care workers? SARs inevitably affect care practices. Therefore, discussions about the future vision of care, including changes to the conceptual framework of care, should not be ignored. In addition, they found justice matters such as distributive justice, the politics of SAR technology, responsibility, recognition, social equality, robot decision-making, and ecological sustainability (Boada et al., 2021).

## 3 ELSI concerning metaverse and XR technologies

Metaverse and XR technologies are among the deepest areas of CAs, as CAs also encompass virtual bodies in cyberspace. Therefore, the findings on ELSI of the metaverse and XR should not be ignored. In many studies, issues on bias, prejudice, and discrimination have been identified as the ELSI of the metaverse. Moreover, misinformation and deepfakes (and deception by them) are seen as major risks with these technologies.

An important legal issue of these technologies is the infringement of intellectual property and copyrights, with particular focus on issues of rights to the data for the machine learning process (Lin and Latoschik, 2022; Benjamins et al., 2023; Kourtesis, 2024; Rudschies and Schneider, 2024; Yasuda, 2025). Ben Chester Cheong discussed issues on rights and responsibilities of avatars in metaverse. Cheong also discussed that potential legal issues in the metaverse include the areas of data protection and privacy, intellectual property rights and personal harm (in terms of harassment and injuries) in the short term. Considering these issues, the previous study suggested that the corporate law framework has potential to become a significant referenced law on this matter. Cheong also examined the possibility of existing intellectual property law as well as consumer protection law to overcome these issues (Cheong, 2022). These discussions provide useful insights into the rights and responsibilities of CA. Concerns about social polarization in the metaverse sphere was pointed out, similar to echo chambers on social media (Benjamins et al., 2023). They have also focused on matters of freedom. For example, the balance between the freedom of expression and harmful content has become a problem. Should virtual attacks on the metaverse be considered criminal offenses? This point is connected to the issues of legal uncertainty and regulatory loopholes. Concerns about social relationships are common in discussions about social robots and HRI. The greater the reality of the metaverse and sophisticated VR, the greater the risk that social relationships will be weakened. In this context, we must consider the increase in disappointment and grief associated with reality and cyberbullying. In addition, Manipulation, which is often seen as an ethical matter and a violation of personal dignity, has also become a problem (Benjamins et al., 2023; Yasuda, 2025).

Governance and security concerns are other important topics in ELSI in relation to metaverses. Protecting data privacy is a major theme because various behaviors can be easily recorded in the metaverse. This has led to issues related to privacy exposure, hacking, and data leakage. Issues surrounding identities have also attracted attention because people can freely create and use their own virtual avatars in the metaverse sphere. However, these avatars sometimes match the identity of the user. Virtual avatars are at risk of being hacked or penetrated. The risks of cybercrime, such as terrorist recruitment and money laundering, should not be overlooked. There are regulatory gaps in this respect. The current legal system cannot cope with the rapid progress in technologies and services (Lin and Latoschik, 2022). In connection with the discussion on governance, the OECD published a policy paper titled *An Immersive Technologies Policy Primer* in March 2025. It highlights the following risks and policy challenges: secure processing and storage of data, risks of biased or misleading interactions in virtual worlds, misinformation and disinformation, harm to democracy, and unequal access to immersive technology due to infrastructural and social inequality (OECD, 2025).

## 4 ELSI concerning exoskeleton and embodied technology

The discussions on ELSI related to exoskeletons and embodied technology focus on dual use, technological accessibility, overdependence of users on the technology, and the legal uncertainty of these technologies (Greenbaum, 2016). How can the misuse and discriminatory use of technology be prevented? This is the central question of these discussions. At the very least, we need to avoid or minimize user dependence on technologies that have negative consequences, such as company bankruptcy or withdrawal. In the legal realm, these technologies should be examined from the perspectives of criminal offenses, torts, product liability, privacy, worker compensation, and worker rights. Furthermore, these technologies blur the boundaries between ability and disability.

In addition, we need to recognize that attitudes towards exoskeletons and embodied technology differ between people with and without prostheses. Consequently, the acceptance of advanced prosthesis wearers is positive. In addition, the abilities of wearers of bionic prostheses are rated highly but not as highly as those of healthy people. However, bionic prostheses are more easily recognized for their functionality and warmth and provide psychological benefits in addition to functional enhancements (Meyer and Asbrock, 2018; Mandl et al., 2022). Interestingly, Mandl et al. pointed out that the term “cyborg” carries with it a negative imaginary (Mandl et al., 2022).

## 5 ELSI on cybernetic avatars

Although few publications currently address ELSI issues related to CAs, research on this topic is gradually progressing. For example, CAs are currently introduced to educational support to children who cannot attend to school. Spoden and Ema (2024) pointed out that there are hurdles of budgets and protection of personal information.

As for the case of DAWN café’s CA “OriHime,” (https://dawn2021.orylab.com/), Ema (2024) found that in certain cases, people mistakenly believed that there was no one inside “OriHime” and made offensive remarks through their observations and interviews with the DAWN café staff. This study revealed new types of problems related to the relationship between humans and robot avatars. Also in social marketing studies, Moriuchi (2023) investigated the attitudes of American consumers toward robotic avatars, focusing on the case of “OriHime” at the DAWN café in Japan. Three types of studies were conducted in which American participants were shown explanations and movies about the robot avatar “OriHime.” In Study 1, the emotional reactions of 11 participants were recorded when they watched a video of a CA in a DAWN café. The result was that positive and joyful emotional reactions dominated. In Study 2, a total of 347 responses were collected, and structural equation modeling (SEM) was carried out to analyze how psychological factors affect the public’s attitude toward the robot avatar “OriHime.” It was also found that an inclusive perspective increased the positive attitude toward “OriHime.” Moreover, the uncanny valley effect had a negative influence on motivation to visit the DAWN café. In Study 3, the attitudes of people with mobility problems were compared with those of people without mobility problems. A total of 334 responses were collected, and the participants’ attitudes toward visiting the DAWN café were examined. It was found that both groups showed a negative uncanny valley effect. In addition, attitudes toward inclusion influenced attitudes toward visiting cafés. People with mobility problems showed a stronger trend where belief in social importance positively influenced their attitude toward visiting the café.

For example, studies on CAs in law have gradually progressed. Scholars have investigated the possibilities of legal representation of CAs (Shimpo, 2024). Shimpo examined the status of CAs based on two categories, “physical CA” and “non-physical CA.” It was pointed out that the legal identification and authentication of a CA differ between the two types. In addition, the legal treatment of identified and anonymous CAs was discussed. In both cases, there is legal uncertainty in the current Japanese legal system. The Cybernetic Being Project (https://cybernetic-being.org/en/) also addresses the legal uncertainty of CAs. It focuses on occupational issues in the use of CAs, particularly robotic avatars. “Guidelines for the Introduction of Avatar Robots in the Workplace 2024: Focusing on Labor Status and International Private Law” (https://cybernetic-being.org/activities/avatarrobot_workdesign_guideline_2024/) have been published. The project examines the laws related to the use of robotic avatars in the workplace, such as the Labor Standards Act, the Labor Contract Act, and the Labor Union Act. In addition, it discusses how working with a robotic avatar allows users to work across their national borders. Considering these ELSI, Nakano et al. (2025) suggested avatar social implementation guidelines based on five common principles: beneficence, non-maleficence, autonomy, justice, and explicability (e.g., Floridi et al., 2018). This ethical guideline idea emphasized the importance of human rights, dignity, sustainability and inclusiveness, wellbeing, trust, protection of privacy, free from discrimination and disparity, democracy, responsibility and accountability, and etc. They also pointed out the importance to examine potential vulnerability in the future society which CA are implemented and cultural adaptation of CA (Nakano et al., 2025).

CAs have been examined from a philosophical perspective. Mizukami (2025) pointed out the philosophical aspects of CA, such as the different perceptions of avatars arising from diverse standards of what constitutes “substantial,” the potential of virtual spaces to pose challenges that go beyond the real world, CA technology, and the new forms of existence and norms for “us.”

## 6 Discussion and conclusion

This review found that ELSI in fields related to CAs includes common themes such as security and safety, data privacy, identity theft, loss of identity, manipulation, intellectual property rights and copyright, addiction, design for accessibility, cyberbullying, risk management in the use of certain services (such as medical services), weakening of social/human relationships, energy issues, environmental issues, balancing freedom of expression, harmful/offensive content, regulatory gaps, the role and limits of human-centered design, accountability, ensuring transparency, distributive justice, widening inequalities, and dual use. These themes can be summarized as those involving protecting human dignity and rights, preventing the harmful effects of technologies on human relationships and professional practices through governance, overcoming legal uncertainty and regulatory gaps, creating a sustainable ecosystem, and considering new conceptual frameworks and the changes brought about by new technologies (Figure 1). These questions are also seen as being inevitable in the ELSI discussions on CAs.
